# Plasma Signaling Factors in Patients With Langerhans Cell Histiocytosis (LCH) Correlate With Relative Frequencies of LCH Cells and T Cells Within Lesions

**DOI:** 10.3389/fped.2022.872859

**Published:** 2022-06-29

**Authors:** Jenée Mitchell, Egle Kvedaraite, Tatiana von Bahr Greenwood, Magda Lourda, Jan-Inge Henter, Stuart P. Berzins, George Kannourakis

**Affiliations:** ^1^Fiona Elsey Cancer Research Institute, Ballarat, VIC, Australia; ^2^School of Science, Psychology and Sport, Federation University Australia, Ballarat, VIC, Australia; ^3^Childhood Cancer Research Unit, Department of Women's and Children's Health, Karolinska Institutet, Stockholm, Sweden; ^4^Center for Infectious Medicine, Department of Medicine Huddinge, Karolinska Institutet, Karolinska University Hospital, Stockholm, Sweden; ^5^Department of Clinical Pathology and Cancer Diagnostics, Karolinska University Hospital, Stockholm, Sweden; ^6^Astrid Lindgren Children's Hospital, Karolinska University Hospital, Stockholm, Sweden

**Keywords:** Langerhans cell histiocytosis (LCH), LCH cells, FoxP3+ regulatory T cells (Treg), mucosal associated invariant T cells (MAIT), active TGF-β, cytokines, T cells

## Abstract

Langerhans cell histiocytosis (LCH) lesions contain an inflammatory infiltrate of immune cells including myeloid-derived LCH cells. Cell-signaling proteins within the lesion environment suggest that LCH cells and T cells contribute majorly to the inflammation. Foxp3+ regulatory T cells (Tregs) are enriched in lesions and blood from patients with LCH and are likely involved in LCH pathogenesis. In contrast, mucosal associated invariant T (MAIT) cells are reduced in blood from these patients and the consequence of this is unknown. Serum/plasma levels of cytokines have been associated with LCH disease extent and may play a role in the recruitment of cells to lesions. We investigated whether plasma signaling factors differed between patients with active and non-active LCH. Cell-signaling factors (38 analytes total) were measured in patient plasma and cell populations from matched lesions and/or peripheral blood were enumerated. This study aimed at understanding whether plasma factors corresponded with LCH cells and/or LCH-associated T cell subsets in patients with LCH. We identified several associations between plasma factors and lesional/circulating immune cell populations, thus highlighting new factors as potentially important in LCH pathogenesis. This study highlights plasma cell-signaling factors that are associated with LCH cells, MAIT cells or Tregs in patients, thus they are potentially important in LCH pathogenesis. Further study into these associations is needed to determine whether these factors may become suitable prognostic indicators or therapeutic targets to benefit patients.

## Introduction

Langerhans cell histiocytosis (LCH) is a rare but likely underdiagnosed disease with approximately 1/200,000 cases per year recorded in children under 15 years. LCH typically presents as one or more inflammatory lesions in any bodily tissue. Lesions comprise myeloid lineage LCH cells that often harbor *BRAFV600E* or other MAPK pathway mutations (1, 2), and whilst the presence of LCH cells is indicative of active disease, lesions characteristically also include a range of other immune cells. Foxp3+ regulatory T cells (Tregs) are typically enriched in patients with LCH (3, 4) and additionally there is a cytokine milieu within lesions that suggests LCH cells and T cells contribute to the localized inflammation (5, 6). This cytokine milieu may further dictate the immune environment, and soluble cell signaling molecules are likely important for immune cell recruitment to lesions.

The clinical outcome for patients with LCH is diverse. Cell infiltration to risk-organs (liver, spleen and hematopoietic involvement) often carries a poor prognosis, and approximately 50% of all patients experience recurrence following the standard of care vinblastine and prednisone therapy. Involvement of the central nervous system may also occur, most commonly including infiltration of the pituitary gland, but a progressive permanent neurodegeneration may also develop. In contrast, untreated lesions in osseous and cutaneous tissue can sometimes resolve spontaneously (7). Given the wide range of clinical outcomes, the presence of a large immune infiltrate, and the concept that lesions can self-resolve, it is likely that the immune system is involved in LCH pathogenesis.

Elevated serum/plasma and cerebrospinal fluid levels of various cytokines and chemokines have been reported and associated with disease extent in LCH and may be important for LCH cell migration and immune cell recruitment to lesions (8–19). Corroborating on the role of the different cytokines and immune cells addressed in these studies, cytokines that typically, although not exclusively, are associated with pro-inflammatory capacity have been detected at higher levels in patients with active disease. Little is known on the relationship between cytokine levels in blood and the relative frequencies on the immune cells in LCH lesions (e.g., LCH cells, Tregs, MAIT cells). Here we investigated whether plasma signaling factors differed between patients with active LCH (AD) and non-active LCH (NAD) (Table 1) in groups closely matched in age (Supplementary Figure S1A), and addressed the link between these factors and relative frequencies of immune cells in LCH lesions.

**Table 1:** Relevant clinical information for the patient cohort assessed in this study.

**Patient code**	Type of sampling: Matched blood/plasma and LCH lesion*or Blood/plasma only	**Specimen description**	**Sex**	**Age at diagnosis**	**Tissues affected**	**Age at specimen**	**Treatment prior to specimen**	**Status at specimen**	**Other**
**A**	Matched	Bone lesion, matched blood/plasma	F	8 months	Bone, skin, lung	8 months	None	AD	BRAF V600E+, multifocal bone, CNS risk lesions
**B**	Blood/plasma only	Blood/plasma	M	7 months	Bone	17 months	Vinblastine, steroids, cytarabine, vincristine	AD	Multifocal, CNS risk lesion
**C**	Matched	Bone lesion, matched blood/plasma	M	2.5 years	Bone, skin	2.5 years	None	AD	Multifocal bone LCH, including CNS risk lesion
**D**	Matched	Bone lesion, matched blood/plasma	M	5 years	Bone	5 years	None	AD	
**E**	Blood/plasma only	Blood/plasma	F	3 years	Bone	5 years	None	NAD	Multifocal
**F**	Matched	Bone lesion, matched blood/plasma	M	7 years	Bone	7 years	None	AD	Mutation in BRAF V600
**G**	Blood/plasma only	Blood/plasma	M	3 years	Lymph nodes, bone, skin, CNS	9 years	Vinblastine, steroids	AD	Diabetes insipidus
**H**	Blood/plasma only	Blood/plasma	F	10 years	Bone	11 years	None	NAD	
**I**	Blood/plasma only	Blood/plasma	M	7 months	Skin, lymph nodes, liver, ears, spleen, bone marrow, intestines, bone	11 years	Vinblastine, steroids, methotrexate, 6-MP, Cladribine, Cytarabine. Modified salvage therapy LCHIV. (no treatment prior to the specimen)	NAD	Non-BRAF mutation
**J**	Blood/plasma only	Blood/plasma	M	10 years	Bone, skin	12 years	Cytarabine, prednisolone and vinblastine (ceased 6 months prior to specimen	AD	Diabetes insipidus, mutation in BRAF V600
**K**	Blood/plasma only	Blood/plasma	F	15 months	Skin, bone, intestines, bone marrow	13 years	Vinblastine, steroids, methotrexate, 6-MP (no treatment prior to the specimen)	NAD	CNS suspicion
**L**	Blood/plasma only	Blood/plasma	F	10 years	Bone	13 years	None	NAD	Unifocal
**M**	Blood/plasma only	Plasma	F	36 years	Bone	37 years	None	NAD	
**N (AD)**	Matched	Pulmonary lesion, matched blood/plasma	M	40 years	Lung	40 years	None	AD	Mild pulmonary fibrosis, smoker
**O**	Blood/plasma only	Blood/plasma	M	41 years	Skin	41 years	Vinblastine, prednisolone	AD	
**N (NAD)**	Blood/plasma only	Plasma	M	40 years	Lung	42 years	Vinblastine, prednisolone	NAD	Mild pulmonary fibrosis, smoker
**P**	Blood/plasma only	Plasma	F	25 years	Bone	42 years	Vinblastine, prednisolone	NAD	Ataxia at time of specimen
**Q**	Blood/plasma only	Plasma	M	39 years	Lung	52 years	Vinblastine, prednisolone	NAD	
**R**	Matched	Skin lesion, matched blood/plasma	F	54 years	Skin	54 years	None	AD	BRAFV600E+
**S**	Blood/plasma only	Plasma	F	60 years	Skin	64 years	Methotrexate, prednisolone	NAD	Leg scarring
**T**	Blood/plasma only	Plasma	M	67 years	Bone, skin	67 years	Short term oral hydroxyurea (not well tolerated)	NAD	
**U**	Matched	Bone lesion, matched blood/plasma	M	68 years	Bone	68 years	Irradiation of a prior lesion in a different location	AD	Diabetes insipidus from age 55

^*^ Matched blood/plasma and LCH lesion are indicated as “Matched”.

## Methods

Lesions and peripheral blood (including plasma) were collected from patients under approval from the Ballarat Health Services and Saint John of God Ballarat Hospital Human Research Ethics Committee and Federation University Australia Human Research Ethics Committee. Patients (or parents/guardians of children where appropriate) provided written, informed consent. Patients were biopsy-diagnosed by pathologists as determined by positive immunohistochemical staining of CD1a and S100 in lesions. Peripheral blood mononuclear cells were isolated from blood, while lesional tissues were digested into single cell suspensions as previously described (20).

A comprehensive range of immune checkpoint molecules, pro-inflammatory chemokines and other cytokines (38 analytes total) were examined using LEGENDplex assays (BioLegend) as per manufacturer's instructions (Table 2). We selected pro-inflammatory chemokines and cytokines because they may contribute to infiltration of inflammatory cells, and soluble immune checkpoint molecules because plasma levels are increasingly shown to be involved in immune regulation.

**Table 2:** Plasma signaling factors analyzed in this study.

**LEGENDplex panel name**	**Human cytokine panel 2**	**Human immune checkpoint panel 1**	**Human pro-inflammatory chemokine panel**
Plasma signaling factors included in the panel	TSLP IL-1α IL-1β GM-CSF IFN-α2 IL-23 IL-12p40 IL-12p70 IL-15 IL-18 IL-11 IL-27 IL-33	sCD25 (IL-2Ra) 4-1BB SCD27 B7.2 (CD86) Free Active TGF-β1 CTLA-4 PD-L1 PD-L2 PD-1 Tim-3 LAG-3 Galectin-9	MCP-1 (CCL2) RANTES (CCL5) IP-10 (CXCL10) Eotaxin (CCL11) TARC (CCL17) MIP-1α (CCL3) MIP-1β (CCL4) MIG (CXCL9) MIP-3α (CCL20) ENA-78 (CXCL5) GROα (CXCL1) I-TAC (CXCL11) IL-8 (CXCL8)

*All plasma samples (see Table 1) were tested for each of the 38 analytes*.

For all flow cytometry experiments, doublets and dead cells were excluded. Viability dye (7-AAD; BD Pharmingen or fixable viability stain 700; BD Horizon), human Fc block (BD Pharmingen) and human antigen specific antibodies (Table 3) were used to identify populations, utilizing gating strategies presented in Supplementary Figure S2. Analyses to determine statistical significance were conducted using GraphPad Prism (GraphPad Software).

**Table 3:** Flow cytometry antibodies used for LCH cell and T cell subset identification.

**Antibody**	**Fluorochrome**	**Clone**	**Company**
CD1a	BV605	SK9	BD Biosciences
CD3	PE-Cy7	UCHT1	BD Pharmingen
CD3	BV650	UCHT1	BD Horizon
CD3	PerCP-Cy5.5	SK7	BD
CD4	BV650	SK3	BD Horizon
CD4	BV711	SK3	BD Horizon
CD4	APC/Fire750	RPA-T4	BioLegend
CD8	APC-Cy7	SK1	BD Pharmingen
CD8	BV510	RPA-T8	BD Horizon
CD8	PE/Cy5	HIT8a	BioLegend
CD11c	PE-CF594	B-ly6	BD Horizon
CD19	BV510	SJ25C1	BD Horizon
CD25	PE-Cy7	M-A251	BD Pharmingen
CD25	BV711	2A3	BD Horizon
CD56	BV786	NCAM16.2	BD Horizon
CD127	BV421	HIL-7R-M21	BD Horizon
CD161	APC	HP-3G10	BioLegend
CD161	PE-Vio770	191B8	Miltenyi Biotec
TCR Vα7.2	FITC	3C10	BioLegend

## Results and Discussion

**Figure 1:**
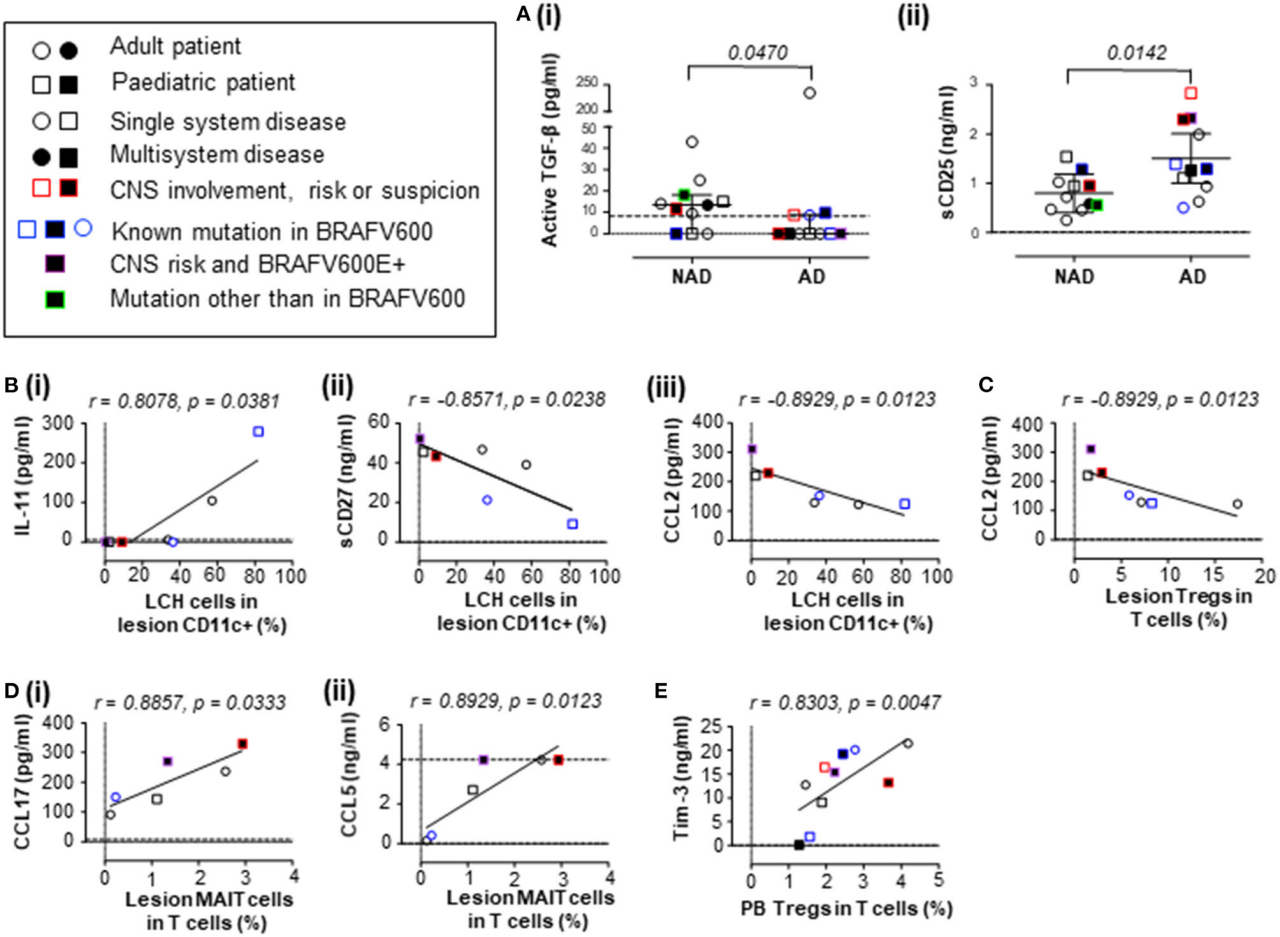
Concentrations of plasma signaling factors in patients with LCH, and their associations with LCH cells and T cell subsets. **(A)** Plasma concentrations of **(i)** active TGF-β (two-tailed unpaired Mann-Whitney test, error bars indicate median + interquartile range) and **(ii)** sCD25 (IL-2Rα; two-tailed unpaired t test with Welch's correction, error bars indicate mean +95% confidence interval) in patients with LCH. **(B)** Correlations between the proportion of LCH cells in lesion CD11c+ cells and plasma concentration of **(i)** IL-11, **(ii)** sCD27 and **(iii)** CCL2 (MCP-1). **(C)** Correlation between the proportion of Tregs in T cells from LCH lesions and plasma concentration of CCL2. **(D)** Correlation between the proportion of MAIT cells in T cells from LCH lesions and plasma concentration of **(i)** CCL17 (TARC) and **(ii)** CCL5 (RANTES). **(E)** Correlation between the proportion of MAIT cells in T cells from peripheral blood from patients with AD and plasma concentration of Tim-3. For **(B–E)**, Spearman's two tailed non-parametric correlation tests were completed. Dashed lines indicate minimum (and maximum for **D.ii**) detectable concentrations as determined by standard curve. For consistency, values below the detectable limit were recorded as zero (dotted lines indicate zero). NAD, non-active LCH; AD, active LCH; CNS, central nervous system; circles represent adult patients, squares represent pediatric patients, open circles/squares represent single system disease, closed circles/squares represent multisystem disease, red borders indicate CNS involvement, risk or suspicion, blue borders indicate known mutation in BRAFV600, purple borders indicate BRAFV600E+ CNS risk lesion and green borders indicate mutation other than BRAFV600.

We unexpectedly found that the active form of transforming growth factor beta (TGF-β) was lower in plasma from a mixed-age cohort of patients with AD when compared to plasma from patients with NAD (Figure 1A.i). This was unexpected because TGF-β was previously reported to be increased in blood from pediatric patients with AD when compared with NAD (9), however it is unclear whether this was free active TGF-β or latency-associated peptide-bound TGF-β, which forms a latent complex. Importantly, our assay tested for free active TGF-β, which has pleiotropic biological effects. It was suggested in one previous report that TGF-β is a potential driver of circulating LCH-like cells *in vivo* (9), and it is well-documented that TGF-β is one of the components able to drive LCH program/state/phenotype in different myeloid cell subsets *in vitro* (21–23). Our finding suggests that we reconsider how TGF-β is involved in LCH pathogenesis. One may speculate that while promoting the pathognomonic LCH program at the site of the lesion, higher levels of circulating active TGF-β specifically in non-active LCH patients may reflect the immunosuppressive nature of TGF-β. The previous study also found that thymic stromal lymphopoietin (TSLP) was elevated in patients with AD (9) and we found a similar trend (Supplementary Figure S1B). In addition, we identified higher levels of soluble CD25 (sCD25) in plasma from patients with AD when compared to plasma from patients with NAD (Figure 1A.ii). It is already established that patients with LCH have elevated serum levels of sCD25, which is associated with disease extent and lower survival rates (12, 17). Our result is consistent with the previous studies investigating sCD25 and supports that sCD25 is associated with disease activity. The mechanistic insights into steps leading to sCD25 elevation in LCH as well as its modulatory capacity on immune cell populations during the active disease remain to be elucidated.

We next aimed to understand whether there were correlations between the levels of plasma signaling factors and the relative frequency of LCH cells. Our study found that the mean concentration of active TGF-β was 13.64 pg/mL in plasma from patients with NAD, consistent with previously reported levels in a control group [3–16 pg/mL (24)]. The study by Carrera Silva et al. suggested that TGF-β and TSLP might drive the plasma induced expression of CD207 in circulating myeloid cells in LCH patients, but LCH-like cells were not detected in the NAD group (9), which we found to have higher levels of active TGF-β than the AD group. Of note, we were only able to detect LCH cells in the lesions, and never in the circulation regardless of disease activity, in line with a recent study including 217 pediatric LCH patients where the whole circulating mononuclear phagocyte compartment was investigated (25). With regard to the LCH program, multiple soluble factors may contribute to the LCH cell phenotype, and LCH themselves are likely to produce high levels of inflammatory cytokines, given their newly described program of senescence and the senescence-associated secretory phenotype (26). We therefore investigated whether there were associations between levels of plasma factors, that may to a certain degree reflect the inflammatory milieu at the site of the lesion, and the proportion of LCH cells in the CD11c+ compartment from plasma donor-matched lesions as measured by flow cytometry (Supplementary Figure S2A).

We did not detect a correlation between LCH cells (*n* = 7) and the concentration of active TGF-β (r = 0.134, *p* = 0.810), TSLP (r = −0.090, *p* = 0.857) or sCD25 (r = −0.571, *p* = 0.200) in plasma. Although patient plasma can drive an LCH-like cell phenotype (9), the plasma levels of active TGF-β and TSLP do not appear to directly affect the proportion of LCH cells within lesions. Investigating associations between lesion LCH cells and other signaling factors, we found that the proportion of LCH cells in lesions correlated with plasma IL-11 (a cancer mediator), soluble CD27 (sCD27; a T cell activator) and plasma CCL2 (MCP-1; monocyte chemoattractant protein-1) (Figure 1B.i–iii). In addition to this study, it is already established that plasma IL-23 and IL-12p40, the two subunits of IL-23 - a well-established driver of chronic tissue inflammation (27), correlate with the proportion of LCH cells in the CD11c+ compartment of LCH lesions (19). The influence of plasma signaling factors on LCH cell phenotype and pathogenesis may be more complex than originally thought, and future studies addressing soluble and cellular immunological phenotypes during AD and NAD in LCH, both at the lesion site and in circulation, are warranted. Here we highlight that several plasma factors correlate with the proportion of LCH cells in lesions that may influence or be influenced by LCH cells.

T cells are also suggested to contribute to the inflammatory environment and we (4, 20, 28) and others (3, 29, 30) have identified abnormalities in several T cell lineages in patients with LCH. Immune dysfunction is suggested in many cancers, and we hypothesize that T cells are also important in LCH pathogenesis. In particular, our group is interested in the role of Tregs due to their elevated frequency in patients with LCH (3) and mucosal associated invariant T (MAIT) cells, due to their lower relative frequency (20). We therefore extended our study to determine whether relationships existed between plasma signaling factors and these LCH-associated T cell subsets. Using flow cytometry, Tregs and MAIT cells were measured relative to the total CD3 population in plasma donor-matched lesions (Supplementary Figures S2B,C). In addition to their association with LCH cells, plasma CCL2 negatively correlated with the proportion of LCH lesional Tregs (Figure 1C). The proportion of MAIT cells in lesional T cells correlated with the plasma CCL17 (TARC; thymus and activation-regulated chemokine) and CCL5 (RANTES; regulated on activation, normal T cell expressed and secreted) (Figure 1D.i,ii). Future studies will be needed to address the role of these factors in relation to Treg and MAIT cell functions/dysfunctions in LCH. Interestingly, we also observed a strong correlation between the proportion of Tregs in T cells from the blood of active LCH patients and plasma Tim-3, that is an emerging immune check point not only in the context of adaptive immune system, but also innate anti-cancer immunity mediated through dendritic cell responses [reviewed in **(author?)** (31)] (Figure 1E).

## Conclusion

This study highlights that active TGF-β is lower in plasma from patients with AD when compared to those with NAD and therefore it is timely to revisit the role of TGF-β in LCH pathogenesis. We also identified several associations between plasma signaling factors and LCH cells, Tregs and MAIT cells in patients with LCH, thus highlighting that these factors may potentially dictate the LCH immune environment or be a by-product of it. Further research is needed to better understand these associations and what role they play in LCH pathogenesis.

## Data Availability Statement

The raw data supporting the conclusions of this article will be made available by the authors, without undue reservation.

## Ethics Statement

The studies involving human participants were reviewed and approved by Ballarat Health Services and Saint John of God Ballarat Hospital Human Research Ethics Committee and Federation University Australia Human Research Ethics Committee. Written informed consent to participate in this study was provided by the participants' legal guardian/next of kin.

## Author Contributions

TB, EK, ML, J-IH, and GK recruited patients with LCH and provided blood, plasma, tissue samples, and corresponding clinical information. JM and GK designed experiments. JM performed experiments, analyzed results, prepared figures, and wrote the manuscript. GK critically revised the manuscript and led the investigation. EK, TB, ML, J-IH, and SB reviewed the manuscript. All authors contributed to the article and approved the submitted version.

## Funding

This work was supported by grants from the Swedish Children's Cancer Foundation, the Swedish Cancer Foundation and Karolinska Institutet.

## Conflict of Interest

The authors declare that the research was conducted in the absence of any commercial or financial relationships that could be construed as a potential conflict of interest.

## Publisher's Note

All claims expressed in this article are solely those of the authors and do not necessarily represent those of their affiliated organizations, or those of the publisher, the editors and the reviewers. Any product that may be evaluated in this article, or claim that may be made by its manufacturer, is not guaranteed or endorsed by the publisher.
