# Supplementary material for: Plasma Signaling Factors in Patients With Langerhans Cell Histiocytosis (LCH) Correlate With Relative Frequencies of LCH Cells and T Cells Within Lesions
Source: Front Pediatr. 2022 Jun 29;10:872859. doi: 10.3389/fped.2022.872859 (PMC9277082; doi:10.3389/fped.2022.872859)

Supplementary Figures

Figure S1. Age range and plasma TSLP concentration for the patient cohort assessed in this study. (A) Age range of the patients with active and non-active LCH (two-tailed unpaired t test with Welch's correction, error bars indicate mean +95% confidence interval). **(B)** Plasma concentration of TSLP in patients with LCH (two-tailed unpaired Mann-Whitney tests, error bars indicate median + interquartile range). Dashed lines indicate minimum detectable concentrations as determined by standard curve. Dotted lines indicate zero. For consistency, values below the detectable limit were recorded as zero. *NAD = non-active LCH, AD = active LCH, circles represent adult patients, squares represent pediatric patients, open circles/squares represent single system disease, closed circles/squares represent multisystem disease, red borders indicate CNS involvement, risk or suspicion, blue borders indicate known mutation in BRAFV600, purple borders indicate BRAFV600E+ CNS risk lesion and green borders indicate mutation other than BRAFV600.*

Figure S2. Gating strategy to identify LCH cells and T cell subsets in patients with LCH. Flow cytometry gating strategy used to identify **(A)** LCH cells ($CD1a^+$ and $CD11c^+$), **(B)** Tregs ($CD3^+CD4^+CD25^+CD127^{low}$), and **(C)** MAIT cells ($CD3^+V\alpha7.2\ TCR^+CD161^{hi}$; left) and $CD8^+CD56^+$ T cells (right) in LCH lesions.

Figure S1

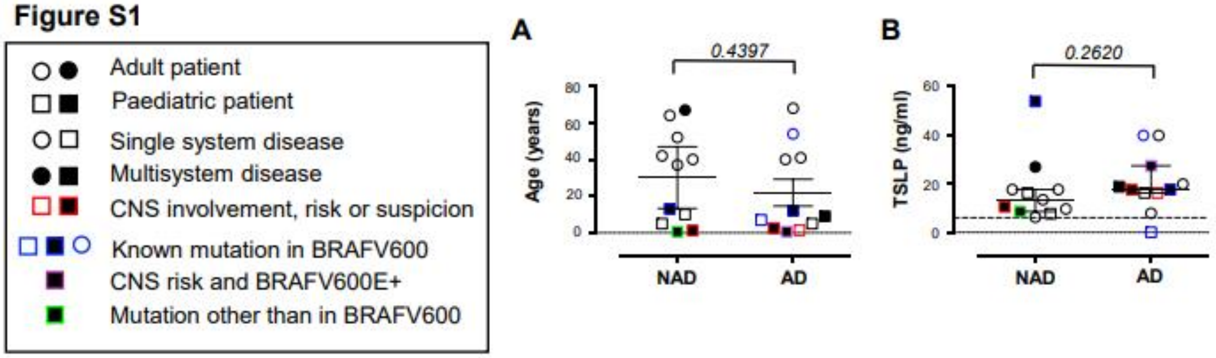

Figure S2

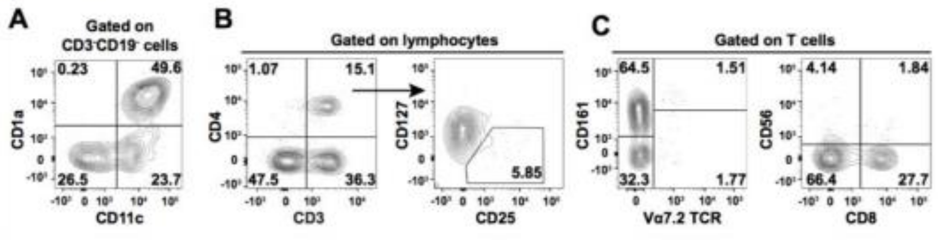

Supplement: Supplementary file 1 [file Data_Sheet_1.PDF]
